# MIP-Based Sensors: Promising New Tools for Cancer Biomarker Determination

**DOI:** 10.3390/s17040718

**Published:** 2017-03-29

**Authors:** Giulia Selvolini, Giovanna Marrazza

**Affiliations:** Department of Chemistry “Ugo Schiff”, University of Florence, Via della Lastruccia 3, Sesto Fiorentino 50019, Italy; giulia.selvolini@unifi.it

**Keywords:** sensor, MIPs, molecularly imprinted polymer, biomarker, cancer, tumor

## Abstract

Detecting cancer disease at an early stage is one of the most important issues for increasing the survival rate of patients. Cancer biomarker detection helps to provide a diagnosis before the disease becomes incurable in later stages. Biomarkers can also be used to evaluate the progression of therapies and surgery treatments. In recent years, molecularly imprinted polymer (MIP) based sensors have been intensely investigated as promising analytical devices in several fields, including clinical analysis, offering desired portability, fast response, specificity, and low cost. The aim of this review is to provide readers with an overview on recent important achievements in MIP-based sensors coupled to various transducers (e.g., electrochemical, optical, and piezoelectric) for the determination of cancer biomarkers by selected publications from 2012 to 2016.

## 1. Introduction

Cancer is defined as a group of diseases involving abnormal cell growth that spreads to other parts of the body beyond their natural boundaries. In the last fifty years, cancer has become one of the leading causes of death worldwide. In Europe in 2012, prostate, lung, colorectal, and bladder cancers were the most common diseases in men; breast cancer was the most common malignancy, followed by colorectal, lung, and corpus uteri cancers in women [1]. If cancer is diagnosed at an early stage, before it has the chance to become too big or spread, it is more likely to be treated successfully. If the cancer has spread, treatment becomes more difficult and generally a person’s chance of survival is much lower. In this context, the need for discovering, developing, and applying new diagnostic tools appears to be evident. New devices have to act as an option for solving the problems within classical clinical diagnostics, such as the need for expensive and sophisticated instrumentation (e.g., computerized axial tomography scan or nuclear magnetic resonance imaging) or qualified personnel, not to mention the long wait time before patients can obtain the analysis results. The precise diagnosis of cancer disease nowadays relies on histological evaluation of tissues using techniques such as enzyme-linked immunosorbent assay (ELISA), radioimmunoassay, immunofluorescence, etc. However, they present some disadvantages as they can be tedious and time consuming and, in addition, they are often unable to provide results in a reasonably short time, making the early diagnosis of cancer more difficult [2]. All these techniques are based on the detection of biomarkers.

Biomarkers are defined both by the Food and Drug Administration (FDA) and the European Union National Institute of Health as a characteristic that is objectively measured and evaluated as an indicator of normal biological processes, pathogenic processes, or pharmacologic responses to therapeutic intervention. The biomarker acts as an indicator of a normal or a pathogenic biological process. It allows for assessing the pharmacological response to a therapeutic intervention. A biomarker shows a specific physical trait or a measurable biologically produced change in the body that is linked to a disease or a particular health condition [3]. In brief, if the concentration of a molecule (i.e., protein, enzyme, microRNA, etc.) varies in dependence to the presence of or during the therapeutic treatment of the disease in biological fluids, we can refer to this molecule as a clinical biomarker [4]. The most common cancer biomarkers are listed in Table 1.

In the field of detection of cancer biomarkers, new bioaffinity sensors have been extensively investigated and were successfully applied as an option for solving all the problems mentioned before, and became useful analytical tools. In fact, biosensors as integrated receptor-transducer devices can overcome the aforementioned difficulties, producing rapidly and selective quantitative or semi-quantitative analytical information. Biomolecular receptors (such as antibodies, enzymes, and histones) are being, however, increasingly replaced with artificial recognition elements, which are often almost as selective as natural ones; in particular, molecularly imprinted polymers (MIPs) are synthetic intelligent materials that can mimic biological recognition well enough to be sometimes called “plastic antibodies” [5].

Here, the MIP-based sensors realized in 2012–2016, coupled with various transducers, for cancer biomarker detection will be reviewed.

## 2. Molecular Imprinting in the Design of Sensors

Molecular imprinting is a process used for preparing affinity polymers for various targets of analytical interest. It involves the formation of a complex between a given target (template) molecule and functional monomers through covalent or non-covalent interactions, which is then subjected to polymerization to form a cast-like shell. The subsequent removal of the template leaves binding sites within the polymer possessing both the correct shape and the correct orientation of functional groups to allow for selective recognition of the imprint species [6,7]. The molecular memory, imprinted on the polymer, is able to selectively bind the target [8,9]. While this work reviews MIPs, it is worth underlining that molecular imprinting is a quite general concept and that there are alternative methods which do not use polymers, such as self-assembled monolayers (SAMs) or molten gallium [10].

MIPs can be prepared for any kind of substance, such as inorganic ions, drugs, nucleic acids, proteins, and so on. They possess a number of advantages in comparison with natural biomolecules [11], as they are stable, specific, low-cost, and easy to prepare and to miniaturize; all these features render them as promising alternatives to the natural bioreceptors used in sensor technology [12].

MIPs are frequently made of acrylic and methacrylic monomers, which usually produce polymers with superior binding and recognition abilities [13]. This is mainly due to the greater flexibility in selecting the appropriate functionalities (because of the large amount of polymerizable compounds that are commercially available) and polymerization protocol (bulk polymerization, grafting, membrane- or particle-shaped, etc.) [14,15]. Other chemical groups currently used in MIP preparation are polyacrylamides [16], polyurethane, siloxanes, and conjugated polymers such as polyaniline and polypyrrole; there are also examples of MIPs produced from inorganic oxides [17]. Recently, Tu et al. have synthesized a series of high-performance MIPs via the photolithographic boronate affinity molecular imprinting approach for immunoassays of glycoproteins in complex real samples [18]. A list of the functional monomers used in MIP-based sensors for cancer marker analysis is reported in Table 2.

These synthetic receptors, able to recognize target molecules by the lock and key mechanism, have been used extensively as sample preparation tools in small molecule analysis. Thus, they have been employed as solid-phase extraction (SPE) sorbents for a wide range of food and environmental targets [19]. However, the growing need for stable and low-cost materials in the design of integrated devices for point-of-care testing (POCT) has led to an increasing interest for the application of MIPs as the molecular recognition element in sensors, exploiting their selective binding. The binding of the target analyte to the MIP can be detected by a change in mass in acoustic sensors, by a change in refractive index in surface plasmon resonance (SPR) sensors, or by electrochemical impedance spectroscopy (EIS) in electrochemical sensors. Numerous analytes which have no optical or electrochemical properties can also be detected by exploiting MIPs’ capacity of generating optical or electrochemical signals in response to the template binding to the functional groups in the imprinting site [20,21]. Very few sensors are described in which MIPs are used as catalysts to produce a detectable electrochemical signal [22,23]. One of the limitations associated with the development of MIP sensors is the difficulty in integrating them with transducers; the most direct and smart way for overcoming this drawback is electro-polymerization, in which the MIP can be synthesized in situ at an electrode surface by simply controlling the layer’s thickness by the amount of charge passed. This approach is particularly attractive for making small devices for clinical diagnostics, environmental control, and for the pharmaceutical industries [9,24,25]. Thin MIP films can also be deposited on a solid support by surface grafting using several types of initiation, such as chemical, UV, or thermal initiation [26]. The synthesis and the immobilization are performed as a one-step procedure through the application of a potential or the exposition to UV light in situ on the detector surface, coated with a mixture of the monomer in an appropriate solvent [27].

For the successful application of MIPs in sensors, it is necessary to improve their binding kinetics, to decrease analysis time, and to remove most of the template. The possibility of designing MIPs at the nanoscale has been demonstrated to have a key effect on these issues by enhancing the surface-to-volume ratio, thus making binding sites more accessible to analytes. Different MIP nanostructures have been prepared and incorporated in the design of sensing devices; among these, MIP nanoparticles (NPs) have been prepared through several strategies and successfully applied in different analytical fields [28,29]. Recently, Mazzotta et al. exploited solid phase synthesis for preparing electroactive MIP NPs by using two electroactive monomers (vinylferrocene and ferrocenylmethyl methacrylate) for the electrochemical detection of the non-electroactive template molecules such as the antibiotic vancomycin [20]. A particular and borderline application of this kind of sensing, as it falls outside the International Union of Pure and Applied Chemistry (IUPAC) definition, is the use of MIP fluorescent core-shell NPs as fluo-tags in a screening method targeting sialic acid [30], whose overexpression had been associated with metastatic cancer [31], proposed by Shinde et al. [32]. The MIP NPs were also applied to four different chronic lymphocytic leukemia cell lines [33].

## 3. Review of the Literature Related to MIP-Based Sensors for Cancer Biomarkers

Nowadays, the need for fast and accurate detection of cancer biomarkers is becoming more and more compelling. The precise design of devices suitable for this purpose plays a crucial role in the effectiveness of the device itself in terms of specificity, cost, limit of detection, and analysis time. In the following sections, MIP-based sensors exploiting different detection techniques for cancer biomarker sensing will be presented (Table 3).

### 3.1. Prostate Specific Antigen

Prostate specific antigen (PSA) is a 30–33 kDa serine protease normally found in serum belonging to the glandular kallikrein protein family (kallikrein 3) and is produced by prostate secretory epithelia. It has several isoforms, with isoelectric points ranging from 6.8 to 7.2 [50,51]. PSA is an approved cancer biomarker from the FDA and is one of the most used cancer biomarkers in the screening and diagnosis of cancer prostate (CaP); the generally accepted cut-off level is 4.0 ng/mL [52]. PSA is not a cancer-specific marker since the PSA level in serum might be elevated in the presence of benign conditions [53]; however, PSA is the only biomarker for the early detection of prostate cancer up to now. Various sensor strategies were realized for PSA analysis.

Ertürk et al. developed a capacitive biosensor with an automated flow injection system for PSA detection [34]. PSA-MIP gold electrodes were prepared in the presence of PSA-modified glass cover slips as protein stamps via UV polymerization of methacrylic acid (MAA) as the functional monomer and ethylene glycol dimethacrylate (EGDMA) as the cross linker. The sensors were characterized by atomic force microscopy (AFM), scanning electron microscopy (SEM), and cyclic voltammetry (CV); immobilized Anti-PSA antibodies on the electrodes for capacitance measurements were also prepared to compare the detection performances of both methods. Real-time detection of PSA was performed with standard PSA solutions by means of an automated flow-injection system, inserting the capacitive electrodes in the electrochemical flow cell and connecting them to the platinum auxiliary and reference electrodes. The capacitance measurement was performed via a current pulse method [54]. A linear range between 0.1 and 10,000 pg/mL with a detection limit of 0.08 pg/mL and a linear range between 1 and 100,000 pg/mL with a detection limit of 0.6 pg/mL were respectively obtained by PSA-MIP sensors and by Anti-PSA biosensors. Selectivity studies were performed against human serum albumin (HSA) and human gamma globulin; as the final step, the reproducibility of the sensors was tested. The proposed sensor was then successfully applied for the detection of PSA in diluted human serum samples.

Jolly et al. achieved the detection of PSA by the development of an aptamer-MIP hybrid receptor amalgamating biomolecular recognition elements and molecular imprinting [35]. A thiolated DNA aptamer with established affinity for PSA was complexed with it prior to being immobilized on the surface of a gold electrode, and then controlled electro-polymerization of dopamine around the complex was performed. It was proposed that after the removal of the PSA template with several washings, the MIP cavity acted synergistically with the embedded aptamer to form a hybrid receptor (apta-MIP) displaying recognition properties superior to that of the aptamer alone. The thickness and root mean squared roughness of the washed and unwashed apta-MIP films on gold-coated glass slides were determined by AFM; a non-imprinted control sensor (apta-NIP) was prepared in the same way, but in the absence of PSA, to compare the performances of the two sensors. The rebinding of PSA to the apta-MIP surface was evaluated by means of electrochemical impedance spectroscopy (EIS). The sensor showed high sensitivity with a linear response from 100 pg/mL to 100 ng/mL and a limit of detection of 1 pg/mL, leading to a sensitivity of around three orders of magnitude higher than the aptamer alone sensor for PSA (LOD 1 ng/mL). The sensor also demonstrated low cross-reactivity with a homologous protein (human kallikrein 2) and low response to HSA.

Rebelo et al. proposed a potentiometric biosensor for the detection of PSA based on a novel plastic antibody designed with charged binding sites to enhance protein binding [36]. Surface imprinting produced this material (C/MIP) over graphene layers to which the protein was first covalently attached. Vinylbenzyl (trimethylammonium chloride) and vinyl benzoate were introduced as charged monomers labeling the binding sites and were allowed to self-organize around the protein. The subsequent polymerization of acrylamide (AAm) as a functional monomer and N,N′-methylenebisacrylamide (MBAAm) was made through the radical route. Neutral MIP (N/MIP) prepared without oriented charges and non-imprinted materials (NIP) obtained without a template were used as controls to check the effect of the charged labels upon the material performance. All the prepared materials were used as ionophores in the membranes of conventional solid-contact carbon electrodes, and the construction of the solid-contact PSA selective electrode was made similarly to that described by Kamel et al. [55]. Overall, the C/MIP sensor showed the best potentiometric response, with a linear range between 2.0–89.0 ng/mL and a limit of detection of 2.0 ng/mL. The effect of foreign species upon a 4 ng/mL solution of PSA was checked for creatinine, urea, glucose, human hemoglobin, and bovine serum albumin (BSA). The performance and the effectiveness of this PSA sensor were also evaluated in biological media with complex compositions, collected from different human prostate cell line cultures, showing that it may represent a useful alternative as a diagnostic tool for PSA determination in biological samples with respect to a commercial ELISA kit [51].

**Figure 1:**
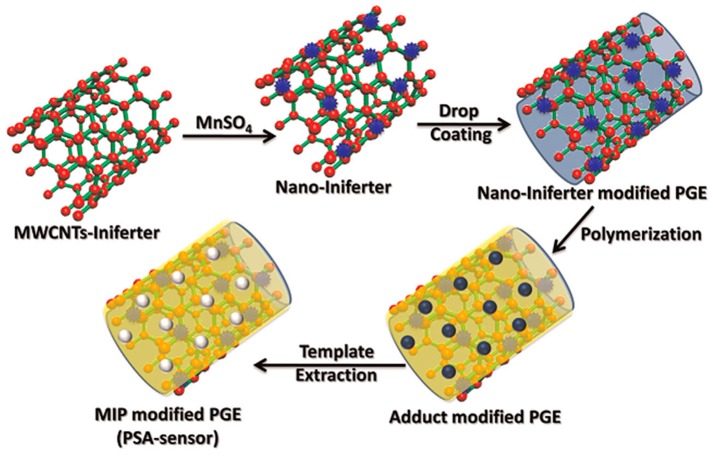
Schematic representation for the fabrication of the PSA sensor. Adapted with permission from [37]. ©2014 Elsevier.

Patra et al. developed a sensitive and selective electrochemical sensor for the detection of PSA combining surface imprinting and nanotechnology [37]. Multi-walled carbon nanotubes (MWCNTs) decorated with MnO_2_ NPs were functionalized with thiol groups to make a nano-iniferter, which was casted onto a pencil graphite electrode (PGE) surface. The nanostructured surface thus obtained was then used as a platform to synthesize a three-dimensional MIP matrix for PSA by controlled radical polymerization of itaconic acid as the functional monomer and ethylene glycol dimethacrylate (EGDMA) as the cross linker in presence of PSA. A schematic representation of the construction of the sensor is reported in Figure 1. The sensor was characterized by Fourier transform infrared spectroscopy (FTIR) and SEM; for the control, a non-imprinted polymer (NIP)-modified PGE was also prepared in the absence of PSA. The qualitative study of PSA was performed by CV and chronocoulometry (CC), whereas quantitative analysis in aqueous and clinical samples was performed by differential pulse and square wave stripping voltammetry (DPSV, SWSV). The detection limit was calculated as low as 3.04 fg/mL and 0.25 fg/mL, respectively. The proposed sensor showed good selectivity against interfering compounds such as insulin, ferritin, urea, albumin, etc., and was thus successfully applied for the determination of PSA in blood serum and urine samples.

Ertürk et al. also reported the development of a micro-contact imprinting based surface plasmon resonance (SPR) biosensor for the real-time detection of PSA from clinical samples [38]. The imprinted chip was prepared in the presence of PSA-modified glass cover slips as protein stamps via UV polymerization of MAA as the functional monomer and EGDMA as the cross linker. The imprinted SPR sensor chip was characterized by AFM, SEM, ellipsometry, dispersive Raman spectroscopy, and FTIR; to determine the selectivity coefficients, a NIP SPR sensor was also developed. PSA detection was performed both in buffered solutions (concentration range 0.1–50 ng/mL, limit of detection 91 pg/mL) and in spiked human serum samples to test the reliability of the system for clinical applications. Selectivity studies were performed using HSA and lysozyme. The reusability of the PSA-imprinted SPR sensor chip was also evaluated by monitoring the change in reflectivity (ΔR) at the same concentration of standard PSA solution (10 ng/mL) and the results indicated that the imprinted sensor chip could be used for PSA detection fifty times with a loss in the activity not exceeding 20% of the first signal value.

### 3.2. Alpha-Fetoprotein

Alpha-fetoprotein (AFP) is a 72 kDa oncofetal protein rarely found in healthy adult organs but is produced by the fetal hepatocytes immediately after birth. The serum AFP level gradually decreases and reaches the 10 ng/mL value within 300 days [56]. Numerous studies have considered AFP as a diagnostic tumor-specific marker for hepatocellular carcinoma (HCC), which has become the fifth most common human cancer with high mortality [57,58], in at-risk patients with a cut-off level of 20 ng/mL [59]. Therefore, AFP was used for serum diagnosis of primary hepatoma [60].

Shen et al. developed a low-cost and easily prepared electrochemical sensor for AFP detection [39]. A MIP-based sensor was fabricated simply by a layer-by-layer coating of chitosan, glutaraldehyde, and the AFP antigen on a glassy carbon electrode, followed by polymerization of AAm as the functional monomer and MBAAm as the cross linker. The sensor was characterized by SEM, CV, and EIS to check the efficiency of the imprinting process; a NIP film electrode was fabricated following the same steps mentioned above without AFP to evaluate the selectivity coefficients. Due to the poor electroactivity of AFP, its detection was achieved by monitoring the redox properties of [Fe(CN)]^3−/4−^ as an electrochemical probe by differential pulse voltammetry (DPV); the method showed a linear range between 0.8–10,000 ng/mL with a limit of detection of 0.096 ng/mL. Selectivity studies were performed with BSA, carcinoembryonic antigen (CEA), and the p24 antigen of human immunodeficiency virus (HIV-p24); reproducibility of the sensors was also tested. In addition, the AFP imprinted sensor was applied successfully for the determination of AFP in a human serum sample.

Karfa et al. proposed a MIP modified electrochemical sensor for the detection of AFP on the surface of specifically designed Ag/AgCl NPs [40]. N-isopropylacrylamide (NIPAAm) was polymerized with N,N′-bisacrylamide as the cross linker in the presence of AFP and cysteine-modified Ag/AgCl nanospheres or nanocubes to explore the role of the shape of NPs both on the binding affinity of imprinted polymers and their electrochemical properties. They found that non-spherical NPs, i.e., cube-shaped, are better platforms for MIP synthesis than conventional spherical NPs: the MIP-cube displayed higher adsorption capacity to the template as compared to the MIP-sphere, which could be attributed to the high surface area of the nanocube in comparison to the spherical NPs. The cube-shaped nanoparticle modified MIP was thus used as a recognition material for the direct sensing of AFP by drop-coating the tip of PGE with a dispersion of the polymer in dimethyl sulfoxide (DMSO). For comparison, a NIP-cube was also fabricated following the same procedure in absence of the template. Detection of AFP was achieved by means of square wave stripping voltammetry (SWSV) both in standard buffered solutions and real samples. The sensor showed a linear range from 0.10 pg/mL to 700 pg/mL with a limit of detection of 24.6 fg/mL. In order to investigate the selectivity of the imprinted sensor, the AFP analysis was also performed in the presence of biological compounds, which are the main components of blood serum, such as arginine, albumin, urea, ascorbic acid, etc., and the data obtained showed that the sensor offered high selectivity and clinical applicability.

**Figure 2:**
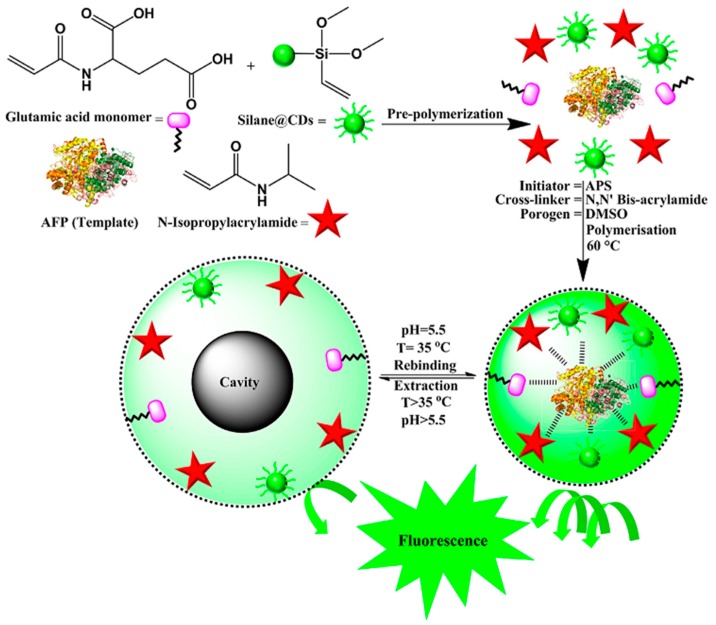
Schematic representation for the synthesis of fluorescence, stimuli-responsive AFP-imprinted polymer. Reprinted with permission from [21]. ©2015 Elsevier.

Karfa et al. reported also the development of an optical sensor for the detection of AFP based on a fluorescent MIP gate with temperature and pH as inputs [21]. This stimuli-responsive fluorescent polymer was synthesized by using three different functional monomers having specific characteristics: 2-acryloylamino-pentanedioic acid (APA), a glutamic acid derivative, for the pH-responsive behavior, NIPAAm as the thermo-responsive monomer, and a vinylsilane modified carbon dot (silane@CDs) to enhance the luminescent property of the imprinted matrix. To provide water-compatibility, MBAAm and ammonium peroxodisulphate were used as the cross linker and the initiator, respectively; a NIP was also prepared to check the role of template binding. The synthesized imprinted polymer had its own fluorescent property, which was enhanced after binding with the template molecule (Figure 2). AFP detection was performed both in aqueous solutions and in real samples; the fluorescence intensity showed a linear response with an increase in concentration of AFP in the range of 3.96–80.0 ng/mL, with a limit of detection of 0.42 ng/mL. The binding of the template to the MIP cavities follow the ‘OR’ logic gate functioning, with temperature and pH as inputs. Along with this, the results obtained were also compared with the commercially available ELISA kit. The authors report that the relative deviation between these two methods was from −1.5% to 1.0%. The proposed sensor also showed good selectivity against interfering compounds such as histidine, urea, albumin, etc., and it was thus successfully applied for the determination of AFP in human blood plasma, serum, and urine samples.

### 3.3. Carcinoembryonic Antigen

Carcinoembryonic antigen (CEA), first described in 1965, is a 180 kDa glycoprotein belonging to the family of immunoglobulins and is mainly involved in cell adhesion [61]. CEA is normally produced in gastrointestinal tissue during fetal development but it is quite absent in the blood of healthy adults; however, the serum levels are raised in some types of cancer. Because the protein was detected only in cancer and embryonic tissue, it was given the name carcinoembryonic antigen, or CEA [62]. It is one of the most used biomarkers, currently employed to measure the course of the progression of colon rectal cancer (CRC) (clinical cut-off 2.5 μg/mL), as suggested by the FDA [63,64,65]. CEA overexpression can also be related to the presence of lung, pancreatic, and breast tumors, and to cancer metastatic processes [66,67].

Moreira et al. developed a low cost and disposable device based on a screen-printed cell with a three electrode configuration (silver working and counter electrodes, silver/silver oxide reference electrode) assembled by printed-circuit board (PCB) technology for CEA detection [41]. The biomimetic material consisted of an imprinted matrix of polypyrrole (PPy), assembled by electro-polymerizing pyrrole on the working electrode surface, in the presence of CEA, followed by protein removal after proteolytic enzymatic activity. A non-imprinted film (NIPPy) was prepared in parallel to assess the contribution of the imprinted sites to the overall analytical response; both films were characterized by FTIR and Raman spectroscopy. The ability of the sensing material to recognize CEA was measured by means of CV, EIS, and square wave voltammetry (SWV) with a Ru(III) solution as the redox probe; the linear response ranged from 0.05 pg/mL to 1.25 pg/mL. Selectivity studies were performed by incubation of the sensor with different molecules; as the final step, reproducibility of the sensor was tested. The results showed that this plastic antibody-based sensor is a promising tool for screening CEA in point-of-care testing (POCT) with good sensitivity and high stability.

### 3.4. Cancer Antigen 125

Cancer antigen 125 (CA-125), also known as mucin 16 (MUC16), is a high molecular weight protein (200 kDa approximately) that in humans is encoded by the MUC16 gene [68,69] and belongs to the mucin family of glycoproteins. CA-125 is produced by the coelomic epithelium and is found on the surface of many ovarian cancer cells [70,71]. Around 90% of women with advanced ovarian cancer have elevated levels of CA-125 in their blood serum, making it a useful tool for detecting ovarian cancer after the onset of symptoms [72]. Elevated levels of CA-125 can be found in individuals affected by other kind of cancers, such as lung or gastrointestinal cancer, or in a number of relatively benign conditions, such as endometriosis [73]. Despite its limited specificity for ovarian cancer, it is the best biomarker for this disease up to now [74].

**Figure 3:**
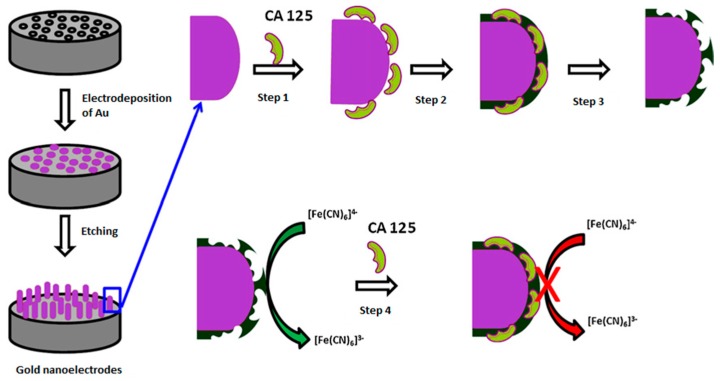
Schematic representation of molecular imprinted protein nanosensor fabrication and template protein detection. Step 1: adsorption of CA-125 onto the nanoelectrode surface; Step 2: electrochemical polymerization of phenol; Step 3: template protein removal; Step 4: CA-125 binding and signal generation. Adapted with permission of [42]. ©2012 Elsevier.

Viswanathan et al. developed a protein imprinted polymer on three-dimensional gold nano-electrode ensemble (GNEE) to detect CA-125 [42]. The nanostructured electrode was prepared by means of electrodeposition of gold within the 50 nm pores of polycarbonate particle track-etched membranes and subsequent electro-polymerization of phenol by CV. The sensor preparation scheme is shown in Figure 3. The sensor then was characterized by SEM to confirm that each template pore was filled with gold. CA-125 detection was performed by monitoring the redox properties of the [Fe(CN)]^3−/4−^ solution by EIS and DPV both in human serum samples in a range of 0.5–400 U/mL with a detection limit of 0.5 U/mL. Selectivity studies were performed with 0.01 mg/mL of HSA, which did not cause significant changes; the reusability of the imprinted sensor was also evaluated by regenerating it up to 20 cycles without a significant loss of activity. The accuracy was examined by comparison of the results obtained by this method with those from the ELISA analysis kit and they were in good agreement.

### 3.5. Nuclear Matrix Protein 22

Nuclear matrix proteins are part of the internal framework of the nucleus and are known to play important roles in DNA replication, transcription of RNA, and regulation of gene expression. The role of nuclear matrix proteins makes them a potential marker for malignant cells which are associated with the abnormal distribution of genetic material and increased mitosis [75]. In particular, nuclear matrix protein 22 (NMP22) is often observed in the urine of patients with urothelial cancer [76] and its use has been therefore approved by the FDA as a monitoring tool for detecting transitional cell carcinoma (TCC) of the bladder [77,78].

Lee et al. achieved the detection of NMP22 by the development of a sensing element comprised of a molecularly imprinted polymer and zinc oxide (ZnO) nanorods to increase the surface area to be coated with MIPs [43]. ZnO nanorods were hydrothermally grown on the screen-printed electrodes, and a solution of poly(ethylene-*co*-vinyl alcohol) in DMSO mixed with template molecules was then added dropwise onto the ZnO nanorods sensing array. For comparison, a NIP film was also fabricated following the same procedure, in the absence of the template; the sensing electrodes were freeze dried before examination by SEM. Detection of NMP22 was performed on real samples by means of CV with [Fe(CN)]^3−/4−^ as the electroactive probe in the range of 128–588 ng/mL. The effects of interference by albumin, lysozyme, and creatinine were also studied and the results showed non-significant changes in the signal; furthermore, although most of the electrodes for measuring blood sugar are disposable, the MIP-based sensor remains stable for a long time and can be stored for reuse [79].

### 3.6. Calcitonin

Calcitonin (also known as thyrocalcitonin) is a 32-amino acid linear polypeptide that is produced in humans primarily by the parafollicular cells (also known as C-cells) of the thyroid gland [80] and was named after its action of reducing blood calcium (Ca^2+^) [81]. The biological activity shown by the calcitonin molecule is due to the presence of a disulphide bridge between residues 1 and 7, eight specific amino acid residues at the N-terminus, and a proline amide moiety at the C-terminus [82]. Calcitonin is used as a tumor marker for medullary thyroid cancer, in which high levels of this hormone is present (clinical cut-off 500 pg/mL) [83].

Patra et al. proposed an imprinted ZnO nanostructure-based electrochemical biosensor for the detection of calcitonin [44]. Firstly, a biocompatible tyrosine derivative as a monomer (2-acryloilamino-3-(4-hydroxy-phenyl)-propionic acid) was grafted covalently onto the surface of a ZnO nanostructure and was then coated onto a vinyl group modified PGE in the presence of calcitonin as the template, EGDMA as the cross linker, a catalyst, and ascorbic acid as the reducing agent. The sensor was characterized by SEM, CV, EIS, and CC; a NIP film electrode was fabricated following the same steps mentioned above without the template molecule. Detection of calcitonin was achieved by means of DPV; the calibration curve shows linearity over a concentration range of 9.99 pg/mL to 7919 ng/mL with a limit of detection of 3.09 pg/mL. Such a sensor can thus predict very small changes in the concentration of calcitonin in the human body and can be considered as cost-effective, renewable, disposable, and reliable for clinical studies, having no such cross-reactivity and matrix effects from real samples.

### 3.7. Bilirubin

Bilirubin (formerly referred to as haematoidin) is a yellow compound that consists of an open chain of four pyrrole-like rings (tetrapyrrole). It is a bioactive molecule produced from hemoglobin metabolism in the blood and is transported to hepatocytes via albumin (as a water soluble complex) and is then excreted into the bile [84,85]. Unconjugated bilirubin (UCB) can accumulate in many tissues, especially in the brain, due to its lipophilic nature, so it cannot be excreted [86]. A high level of UCB in the blood can cause hepatic or biliary tract dysfunction [87], so the bilirubin level in serum is a significant index for liver function and was recently identified and validated as a lung-cancer biomarker by the American Association for Cancer Research [88,89].

**Figure 4:**
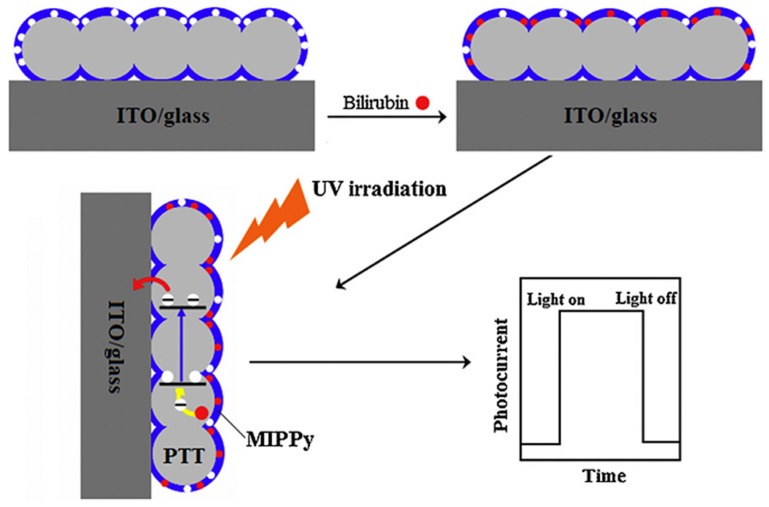
Schematic illustration for the detection mechanism of the bilirubin photoelectrochemical sensor. Reprinted with permission from [45]. ©2015 Elsevier.

Zhang et al. reported the detection of bilirubin through a photoelectrochemical sensor based on porous transparent TiO_2_ (PIT) and molecularly imprinted polypyrrole (MIPPy) [45]. The PIT was fabricated by a sol-gel process in which a solution of titanium tetraisopropoxide (TTIP) in ethanol and hydrochloric acid was deposited on an indium tin oxide (ITO) glass electrode. The MIPPy/PIT/ITO/glass electrode was prepared by polymerizing pyrrole in the presence of bilirubin by ultrasonic irradiation in a nitrogen atmosphere and the subsequent removal of the template molecule (Figure 4). The sensors were characterized by ultraviolet-visible spectroscopy, SEM, FTIR, and X-ray diffraction; for comparison, the non-imprinted PPy-modified electrode was prepared using the same procedure without bilirubin. The ability of the biomimetic material in recognizing bilirubin was achieved by measuring the photocurrent generated due to the photocatalytic oxidization of bilirubin following UV irradiation in a conventional three-electrode electrochemical cell (formed by MIPPy/PIT/ITO/glass as the working electrode, saturated calomel electrode as the reference electrode, and platinum wire as the auxiliary electrodes). The photocurrent was linearly dependent on the bilirubin concentration in the range of 0.03–28 μM with a limit of detection of 0.001 μM. The sensor exhibited outstanding selectivity when used in coexisting systems containing various interferents with high concentrations (such as biliverdin, cholesterol, testosterone, and HSA); the sensor was also applied in real samples.

Çiçek et al. developed a quartz crystal microbalance (QCM) sensor comprising a gold QCM sensor on whose surface was synthesized an imprinted poly-(2-hydroxyethyl methacrylate-N-methacryloil-L-tryptophan methyl ester) (PHEMATrp) nanofilm for bilirubin detection in human plasma [46]. Firstly, bilirubin was pre-complexed with the functional co-monomer of MATrp and the product was mixed with hydroxyethyl methacrylate (HEMA); then, the solution was poured onto the surface of the allyl mercaptan functionalized QCM sensor and was subjected to UV polymerization. A non-imprinted QCM chip (NIP-QCM) was also prepared through the same procedure without using bilirubin to test the efficiency of the imprinting process; the bilirubin imprinted and the non-imprinted QCM sensor surfaces were characterized by Fourier transform infrared spectroscopy-attenuated total reflectance (FTIR-ATR), contact angle measurements, ellipsometry, and AFM. The imprinted QCM sensor showed linearity for the concentration range of 1–50.0 μg/mL with a limit of detection of 0.45 μg/mL. Cholesterol, estradiol, and biliverdin, which are similar in size/shape and molecular weight to bilirubin, were used for selectivity studies; the imprinted sensor was also used to detect total bilirubin in human blood plasma.

### 3.8. Neopterin

Neopterin, a pteridin of low molecular mass (253 g/mol), is a catabolic product of guanosine triphosphate (GTP), a purine nucleotide. Neopterin is considered to be a biomarker of a pro-inflammatory immune response. The increased neopterin concentration in body-fluids, such as serum or urine, indicates cellular immunity activation [90] and has been observed in diseases such as viral and bacterial infections [91,92] including human immunodeficiency virus (HIV) [93], and Lyme disease, parasites such as malaria [94], autoimmune diseases such as rheumatoid arthritis [95], malignant tumor diseases [96], and many other pathologies.

Sharma et al. reported the development of a potentiometric sensor for neopterin detection using an electrochemically synthesized MIP as the sensing element [47]. Preparation of the MIP film involved electrochemical co-polymerization of 2,2′-bithiophene-5-boronic acid and 2-(cytosin-1-yl)ethyl *p*-bis(2,2′-bithien-5-yl)methylbenzolate as functional monomers in the presence of 2,4,5,2′,4′,5′-hexa(thiophene-2-yl)-3,3′-bithiophene as the cross linker and with a neopterin template on the 1-mm diameter platinum disk electrode. A NIP film was synthesized by exploiting the same procedure in the absence of the template molecule for comparison; the morphology of both the electrodes was characterized through AFM. The open-circuit potential (OCP) based transduction under flow-injection analysis conditions (using Pt wire as the auxiliary and Ag/AgCl as the reference electrodes) determined neopterin in the concentration range of 0.15–2.5 mM (38–633 μg/mL) with a limit of detection of 22 μM (5.6 μg/mL). The sensor successfully discriminated the interferences including the 6-biopterin and pterin structural analogs of neopterin, as well as glucose and creatinine; moreover, neopterin in serum samples was detected.

### 3.9. Modified Nucleosides

Modified nucleosides are produced at the post-transcriptional level by chemical modifications of normal nucleosides (e.g., alkylation of the heterocycle, methylation of the sugar, reduction, etc.) [97] liberated during the RNA turnover. The modified nucleosides do not fit into the macromolecular nucleic acid structure, thus they cannot be reutilized for the de novo RNA synthesis and circulate freely in blood before elimination in urines by the same excretion pathway than normal nucleosides [98]. Furthermore, every disorder of RNA turnover increases the level of modified nucleosides and is strictly associated with many types of cancer. Consequently, an increased excretion of abnormal amounts of modified nucleosides in the urine of cancer patients can be observed. Therefore, modified nucleosides are widely regarded as potential tumor biomarkers [99,100]. Recently, Zhang et al. have reported that the intracellular fluctuations of six endogenous nucleotide levels (AMP, UDP, CTP, ATP, UTP, and GMP) were significantly higher in cancer cells [101]. Moreover, the adenine nucleotides are signaling molecules that are related to the modulation of immune responses in cancers [102]. Thus, intracellular nucleotides are considered as potential biomarkers in tumor cells, especially for ATP and UTP. Moreover, oxidative damage to DNA has also been implicated in the pathophysiology of a wide variety of human diseases including cancer [103]. Because the reactive oxidants are not suitable for analysis, oxidized nucleosides like 8-hydroxy-2′-deoxyguanosine (8-OHdG) are used as biomarkers for DNA oxidative damage [104], and consequently for carcinogenesis.

Dejous et al. developed a diagnosis tool for the detection of urinary modified nucleosides based on an acoustic wave (Love wave, LW) biosensor and MIPs as a recognition material [48,105]. The template adenosine-5′-monophosphate (AMP), chosen as a model for nucleosides, was added to a solution of AAm and 2-(dimethylamino) ethyl methacrylate (DMAEM) as the functional monomer in DMSO in the presence of EGDMA as the cross linker. The obtained MIP solution was spin-coated onto the surface of a silane functionalized surface acoustic wave sensor and was subjected to UV polymerization. The obtained sensor was characterized through mechanical and optical profilometry, as well as with SEM; a NIP was prepared similarly for the control experiments. Real-time detection of AMP was performed in aqueous media by associating the acoustic delay-line to a microfluidic chip of polydimethylsiloxane (PDMS). A detection limit of 1 ppm was obtained. Specificity tests were also performed in this dynamic mode using three similar nucleotides adenosine-3′-monophosphate (3AMP), cytidine-5′-monophosphate (CMP), and 2-phosphono methoxypropyl adenine (PMPA). The obtained results showed that LW devices functionalized with MIP films and combined with a microfluidic chip are able to detect, in real-time and with good specificity, nucleoside analogues in low concentrations (100–600 ppm).

Martins et al. proposed an innovative electrochemical sensor assembly to monitor urinary 8-OHdG down to the pmol/L level [49]. The sensing film of the sensor consisted of a MIP layer for 8-OHdG assembled on a monolayer of 3-mercapto-1-hexanol of a previously modified bare gold electrode surface through electro-polymerization of phenol as the functional monomer combined with the template molecule. Each chemical modification step on the gold electrode was characterized by FTIR, Raman spectroscopy, and SEM. Control experiments were carried out with sensors prepared by following the same procedure but without the presence of the template molecule. Detection of 8-OHdG was achieved by means of CV and EIS in a linear range from 0.1 pg/mL to 100 pg/mL. The interfering species (e.g., uric acid, citric acid, and glucose) were tested and good selectivity towards the target molecule was obtained.

## 4. Current Trends and Future Perspectives

Over the last decade, we have witnessed a tremendous amount of research activity in the area of biosensors, since one of their most important applications is the POCT. In the field of cancer biomarker detection, it is desirable to have the highest selectivity possible. This is important in order both to understand the kind of disease and to recognize the clinical use of the detected biomarker. To reach this important goal, MIP-based sensors are constructed by exploiting different types of molecular imprinting techniques, such as bulk imprinting and surface imprinting. In the first approach, a template molecule is imprinted as a whole in the polymer matrix and it needs to be wholly removed from the obtained material after polymerization. The use of whole proteins as a template (as in the case of cancer biomarkers) will increase the accuracy; however, the imprinted sites formed by larger structures might also be attractive for smaller polypeptides which result in cross-reactivity and reduced selectivity. These drawbacks can be overcome in surface imprinting, in which less template molecules are used as compared to those in the bulk approach (because the coating step is limited to the surface), but due to this the recognition sites are more easily accessible with favorable binding kinetics [106].

The early diagnosis of cancer disease is a crucial point in the development of a therapy capable of increasing the survival rate of patients. The clinical use of protein biomarkers for the differentiation of healthy and disease states, and for monitoring disease progression, requires the measurement of low concentrations of proteins in complex samples. The majority of proteins which play an important role as biomarkers in cancer, as well as in neurological disorders and in the early stages of infection, are thought to circulate in the range 10^−16^–10^−12^ M [107], which are very low values to detect even by using a high sensitive method. The developed MIP-based sensors are not very sensitive for applying in efficient early diagnosis, but they can be employed in the screening and monitoring stages of the disease.

In clinical diagnosis, the detection of one biomarker cannot provide sufficient clinical information for all of the various cancer-related diseases, and that obtained from biomarkers is often related to the stage of tumorigenesis, the monitoring of treatment, and the state of the patient. Therefore, it is important to develop microchip systems with high multiplexing capabilities for the detection of a panel of biomarkers such as a fingerprint for cancer.

Compared to the current state-of-the art protein detection, significant advances should be made in terms of the sensitivity, selectivity, and multiplexing capacity for MIP-based sensors.

The analytical potential of sensors in the medical diagnostics field still has to be enhanced by the demonstration of their applicability to real sample analysis. Only a few MIP-based sensors were applied in clinical samples, and their validation with established methods is missing in most cases.

Despite the increasing interest and the extensive research performed on the development of biosensors in several fields (e.g., for clinical, food, and environmental applications), only a small number of these devices are commercially available [108]. The commercial success of biosensors, in contrast to the glucose sensor, has been partially hindered by the absence of appropriate biological receptors that are inexpensive to produce and stable for storage. This problem becomes particularly important when designing devices for needs in medical diagnostics. MIPs may provide a sustainable alternative to solving these problems. Since MIP-based sensors applied in the clinical field are still developing, there are many more steps that need to be done to achieve the construction and marketing of a MIP-based sensor device. Therefore, validated, next-generation diagnostic devices based on sensor technology with the integration of sampling, processing, and analysis to address the most common diagnostic problems are greatly needed.

## 5. Conclusions

In this review, the most recent developments in the design of MIP-based sensors for cancer biomarker detection were reviewed. MIPs represent a promising sensing element combining high affinity for target molecules, low cost, stability, and greater flexibility in choosing the monomers and the polymerization protocol. The coupling of these recognition elements with nanoscale materials offers an enhancement in the binding selectivity as well as in the sensitivity, and improves the development of highly-versatile diagnostic devices ready to be used in clinical analysis directly at the patients’ bedside.

## Figures and Tables

**Table 1:** The most common biomarkers used to detect different cancer types and their clinical use.

Biomarker	Cancer Type	Clinical Use
NMP22	Bladder	Screening and monitoring
Modified nucleosides		Diagnosis, screening, and monitoring
CA-125	Breast, Ovarian	Monitoring
CEA	Colon	Monitoring
Modified nucleosides		Diagnosis, screening, and monitoring
AFP	Liver	Diagnosis
Bilirubin		Diagnosis
Bilirubin	Lung	Diagnosis
Neopterin		Diagnosis and monitoring
PSA	Prostate	Screening and monitoring
Calcitonin	Thyroid	Diagnosis, prognosis, and screening

NMP22: nuclear matrix protein 22; CA-125: cancer antigen 125; CEA: carcinoembryonic antigen; AFP: alpha-fetoprotein; PSA: prostate specific antigen.

**Table 2:** Most common monomers and polymerization procedure applied in MIP-based sensors for cancer biomarker analysis.

Monomer(s)	Cross Linker	Polymerization Procedure	Biomarker
MAA	EGDMA	UV	PSA
Dopamine	–	Electrochemical	PSA
AAm	MBAAm	Radical	PSA, AFP
Itaconic acid	EGDMA	Thermal	PSA
NIPAAm	BAAm	Thermal	AFP
APA + NIPAAm + silane@CDs	MBAAm	Thermal	AFP
Pyrrole	–	Electrochemical	CEA
	–	Ultrasonic	Bilirubin
Phenol	–	Electrochemical	CA-125, modified nucleosides
Ethylene + vinyl alcohol	–	Thermal	NMP22
AAHPhPA	EGDMA	Thermal	Calcitonin
MATrp + HEMA	–	UV	Bilirubin
BTBA + CEBTMB	HTBT	Electrochemical	Neopterin
AAm + DMAEM	EGDMA	UV	Modified nucleosides

MAA: methacrylic acid; EGDMA: ethylene glycol dimethacrylate; AAm: acrylamide; MBAAm: N,N′-methylenebisacrylamide; NIPAAm: N,N′-isopropylacrylamide; BAAm: N,N′-bisacrylamide; APA: 2-acryloylamino-pentanedioic acid; silane@CDs: vinylsilane modified carbon dot; AAHPhPA: 2-acryloylamino-3-(4-hydroxyphenyl)-propionic acid; MATrp: N-methacryloyl-L-tryptophan methylester; HEMA: hydroxyethyl methacrylate; BTBA: 2,2′-bithiophene-5-boronic acid; CEBTMB: 2-(cytosin-1-yl)ethyl p-bis(2,2′-bithien-5-yl)methyl benzolate; HTBT: 2,4,5,2′,4′,5′,-hexa(thiophene-2-yl)-3,3′-bithiophene; DMAEM: 2-(dimethylamino)ethyl methacrylate; PSA: prostate specific antigen; AFP: alpha-fetoprotein; CEA: carcinoembryonic antigen; CA-125: cancer antigen 125; NMP22: nuclear matrix protein 22.

**Table 3:** Analytical parameters of MIP-based sensors for cancer biomarker detection.

Biomarker	Detection Technique	Linear Range	LOD	Reference
PSA	Electrochemical (capacitance)	0.1–10,000 pg/mL	0.08 pg/mL	[34]
	Electrochemical (EIS)	100 pg/mL–100 ng/mL	1 pg/mL	[35]
	Potentiometry	2.0–89.0 ng/mL	<2.0 ng/mL	[36]
	Electrochemical (SWSV)	–	0.25 fg/mL	[37]
	Electrochemical (DPSV)	–	3.04 fg/mL	[37]
	Optical (SPR)	0.1–50 ng/mL	91 pg/mL	[38]
AFP	Electrochemical (DPV)	0.8–10,000 ng/mL	0.096 ng/mL	[39]
	Electrochemical (SWSV)	0.10–700 pg/mL	24.6 fg/mL	[40]
	Optical (fluorescence)	3.96–80.0 ng/mL	0.42 ng/mL	[21]
CEA	Electrochemical (CV, EIS, SWV)	0.05–1.25 pg/mL	–	[41]
CA-125	Electrochemical (EIS, DPV)	0.5–400 U/mL	0.5 U/mL	[42]
NMP22	Electrochemical (CV)	128–588 ng/mL	–	[43]
Calcitonin	Electrochemical (DPSV)	9.99 pg/mL–7919 ng/mL	3.09 pg/mL	[44]
Bilirubin	Photoelectrochemical	0.03–28 μM	0.001 μM	[45]
	Piezoelectric (QCM)	1–50 μg/mL	0.45 μg/mL	[46]
Neopterin	Potentiometry	0.15–2.5 mM	22 μM	[47]
Modified nucleosides	Piezoelectric (acoustic wave)	–	<1 ppm	[48]
	Electrochemical (CV, EIS)	0.1–100 pg/mL	–	[49]

PSA: prostate specific antigen; AFP: alpha-fetoprotein; CEA: carcinoembryonic antigen; CA-125: cancer antigen 125; NMP22: nuclear matrix protein 22; EIS: electrochemical impedance spectroscopy; SWSV: square wave stripping voltammetry; DPSV: differential pulse stripping voltammetry; SPR: surface plasmon resonance; DPV: differential pulse voltammetry; CV: cyclic voltammetry; SWV: square wave voltammetry; QCM: quartz crystal microbalance.
